# Potential Role of *Hepatozoon canis* in a Fatal Systemic Disease in a Puppy

**DOI:** 10.3390/pathogens10091193

**Published:** 2021-09-14

**Authors:** Andrea De Bonis, Mariasole Colombo, Rossella Terragni, Barbara Bacci, Simone Morelli, Marika Grillini, Massimo Vignoli

**Affiliations:** 1 Faculty of Veterinary Medicine, University of Teramo, 64100 Teramo, Italy; andrea.debonis93@gmail.com (A.D.B.); mcolombo@unite.it (M.C.); 2 Clinica Veterinaria Pet Care, via Marzabotto 1/2 M-N, 40133 Bologna, Italy; terragni.rossella@gmail.com; 3 Department of Veterinary Medical Sciences, University of Bologna, Ozzano dell’Emilia, 40064 Bologna, Italy; barbara.bacci@unibo.it; 4 Department of Animal Medicine, Production and Health, University of Padua, 35020 Legnaro, Italy; marika.grillini@phd.unipd.it

**Keywords:** *Hepatozoon* *canis*, dog, skin, liver

## Abstract

Canine hepatozoonosis caused by *Hepatozoon* *canis* is an emerging disease in Europe. Clinical pictures vary from subclinical to life-threatening and non-specific clinical signs are predominantly reported. A 2-month-old female puppy originating from Southern Italy was adopted and moved to Northern Italy. Then, the dog was brought to a local veterinary practice for gastrointestinal signs, migrating lameness and pruritic dermatitis, and then tested positive for *Hepatozoon* spp. gamonts at the blood smear. After treatment with imidocarb dipropionate and doxycycline, the dog showed an initial clinical improvement. However, gastrointestinal signs recurred, and diffuse superficial pyoderma appeared on the thoracolumbar region, along with fever, lethargy, and weight loss. Eight months from the first onset of clinical signs, the dog was referred to a veterinary clinic and subjected to complete blood count, urine and fecal analysis, along with abdominal ultrasonography, whole-body CT and gastroduodenal endoscopy. Skin biopsies and blood samples were subjected to a PCR-coupled sequencing protocol, which scored both positive for *H. canis*. Alterations were consistent with a pre-existing cholangiohepatitis and multiple acquired extrahepatic shunts secondary to portal hypertension. The dog was euthanatized due to a clinical worsening two months later. The potential role of *H. canis* in the systemic disease observed, clinic-pathological findings and epizootiological implications are discussed.

## 1. Introduction

Hepatozoonosis is an emerging vector borne disease (VBD) of both dogs and cats, caused by different species belonging to the genus *Hepatozoon* [1,2]. Canine hepatozoonosis is caused by *Hepatozoon canis* that infects dogs worldwide, and *Hepatozoon americanum* that has been reported, to date, only in the Americas [1,3,4,5]. Dogs are intermediate hosts of *Hepatozoon* spp. and become infected via the ingestion of infected ticks (definitive hosts) harboring mature oocysts. The sporozoites released in the intestine penetrate the gut wall and reach, via lymphatic and blood circulation the target organs, i.e., bone marrow, spleen, lymph nodes, liver, kidney and lungs, where the merogony occurs [1]. The micromerozoites released from mature meronts invade the neutrophils and monocytes, where they mature into gamonts. Ticks become then infected ingesting gamonts contained in the leukocytes during a blood meal on a parasitaemic vertebrate [1].

In Europe, the main vector of *H. canis* is the brown dog tick *Rhipicephalus sanguineus* in which it is transmitted transstadially [5,6,7]. Although the ingestion of an infected tick is the most common source of infection, transplacental transmission and carnivorism have been reported as alternative routes [1,8].

Clinical pictures range from subclinical to severe signs, and non-specific clinical signs, e.g., lethargy, anorexia, weight loss, lymphadenopathy and fever are predominant, while life-threatening conditions may occur in (i) dogs co-infected by other vector-borne pathogens (e.g., *Ehrlichia* spp., *Anaplasma* spp., *Leishmania infantum, Babesia canis*), (ii) immunocompromised animals and (iii) puppies [1,9,10,11]. Skeletal pain, sub-acute periostitis, gastrointestinal and respiratory signs, oral, skin and ocular lesions (i.e., glaucoma and uveitis) have been associated with *H. canis* [1,12,13,14,15]. The most common hematobiochemical alterations are non-specific, i.e., anemia, thrombocytopenia, leukocytosis, hyperproteinaemia with polyclonal hyperglobulinaemia and hypoalbuminaemia, increased creatine kinase, and alkaline phosphatase activities [1,16]. Post-mortem findings include pneumonia, hepatitis, and glomerulonephritis [1]. 

Given the emergence of canine hepatozoonosis in European regions [5,17], knowledge on its epidemiology and clinical features should be implemented. The present report describes a fatal systemic disease in a young dog infected with *H. canis*. Epizootiological and clinic-pathological implications are discussed.

## 2. Case Details

### 2.1. Clinical Case

#### 2.1.1. Visit 1 (2 Months Old)

A 2 month old female mixed-breed puppy originating from Southern Italy was adopted and moved to Northern Italy. The dog received prophylactic treatment for ectoparasites with afoxolaner 5 mg/kg PO and it was regularly vaccinated. Then, the puppy was brought to visit to a local veterinary practice for the onset of diarrhea, vomiting, migrating lameness, and skin lesions that appeared as crusted lesion. The latter were localized mainly in the dorsal portions of the neck and thorax. The animal was initially subjected to complete blood count (CBC), to the evaluation of basal cortisol concentration and to abdominal ultrasonography, along with a fine needle aspiration of the spleen. The dog was also tested for antibodies towards *Ehrlichia canis/Ehrlichia ewingii*, *Anaplasma phagocytophilum*/*Anaplasma platys*, *Borrelia burgdorferi* and antigens of *Dirofilaria immitis* using SNAP 4Dx^®^ (IDEXX) and for *L. infantum* antibodies (IFAT). *Hepatozoon* spp. gamonts were detected at the blood smear and the dog was treated with doxycycline (10 mg/kg SID PO) and imidocarb dipropionate (6 mg/kg) every two weeks, until negativization of the blood smear [18].

#### 2.1.2. Visit 2 (5 Months Old)

After an initial improvement, diarrhea and vomiting recurred 3 months later and diffuse superficial pyoderma appeared on the thoracolumbar region, along with fever, lethargy, and weight loss. The dog was subjected to complete haemato-biochemical examination, to copromicroscopic analyses (i.e., Baermann test, floatation, and SNAP Giardia^®^-IDEXX), and to cutaneous biopsies. The dog was then treated with cefazoline (20 mg/kg q8 EV) for skin lesions and subjected to several diet trials.

#### 2.1.3. Visit 3 (10 Months Old)

After a new initial improvement, 4 months later the clinical pictures worsened again with the onset of anorexia, diarrhea, and vomiting. Then, the animal was referred to a veterinary clinic in Bologna, Italy, and it was again subjected to complete haemato-biochemical examination, urine analysis, coagulation test, pre-/post-prandial bile acids test, leptospirosis MAP test, to a further *L. infantum* IFAT, abdominal ultrasonography, whole-body CT, gastrointestinal endoscopy with hepatic, gastric, duodenal, and cutaneous biopsies. Skin biopsies and blood samples were subjected to a PCR-coupled sequencing protocol for *Hepatozoon* spp. [19,20]. The dog was treated with maropitant (1 mg/kg) SID orally, S-adenosil methionine 200 mg SID orally, co-amoxiclav 20 mg/kg BID orally, Ursodeoxycholic acid 10 mg/kg SID orally, Vitamin B12 500 mcg SC once every 7 days. The clinical conditions of the dog continued to worsen (i.e., lethargy, anorexia, and ascites) and it was euthanized 2 months later.

### 2.2. Findings of the Exams Performed at 2 Months Old

The haemato-biochemical exam showed a mild neutrophilic leukocytosis, mild microcytic normochromic anemia, the basal cortisol was mildly increased (Table 1). The dog tested negative to SNAP 4Dx^®^ and to *L. infantum* antibodies. The fine needle aspiration of the spleen showed an extramedullary hemopoiesis with occasionally neutrophils phagocyting parasite stage compatible with *Hepatozoon* spp.

### 2.3. Findings of the Exams Performed at 5 Months Old

The haemato-biochemical showed a mild microcytic normochromic anemia, mild hypoproteinemia (Table 1), while the remaining chemistry was unremarkable, copromicroscopic exams and SNAP Giardia^®^ were negative. Biopsy of the skin showed a perivascular dermatitis and pyoderma with intralesional bacteria.

### 2.4. Findings of the Exams Performed at 10 Months Old

The results of the exams repeated 5 months later showed moderate thrombocytosis, moderate hypoalbuminemia, mild hypoproteinemia, severe hypocobalaminaemia, mildly decreased folates, severely increased ALT and ALP, moderately increased AST, markedly increased canine TLI, and moderately increased pre- and post-prandial bile acids. Bilirubinuria and urobilinogen were detected in the urine analysis. Mildly increased aPTT was found at the coagulation test (Table 1). The leptospirosis MAP test was negative. The dog was still negative for *L. infantum* antibodies at IFAT.

#### 2.4.1. Computed Tomography

**Figure 1:**
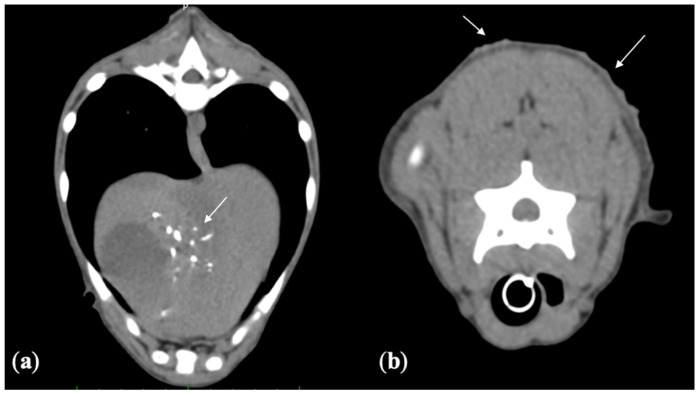
(**a**) Pre-contrast transverse CT image. Multiple mineralization in the biliary ducts (white arrow). (**b**) Pre-contrast transverse CT image. In the dorsal portion of the skin a diffuse irregularity and hyperdense areas (white arrows) are evident.

The whole-body CT examination was taken with a multidetector CT (Optima 540 GE, Milwaukee; WI, USA), and showed multiple mineralizations at the level of the intrahepatic ducts with concurrent dilation of the common bile duct. A severe thickening of the duodenum, most likely consistent with a pre-existing cholangiohepatitis, was detected. Diffuse irregularity and hyperdense areas on the skin were compatible with diffuse dermatitis (Figure 1). Multiple acquired extra hepatic shunts secondary to portal hypertension were also observed.

#### 2.4.2. Ultrasound (US)

**Figure 2:**
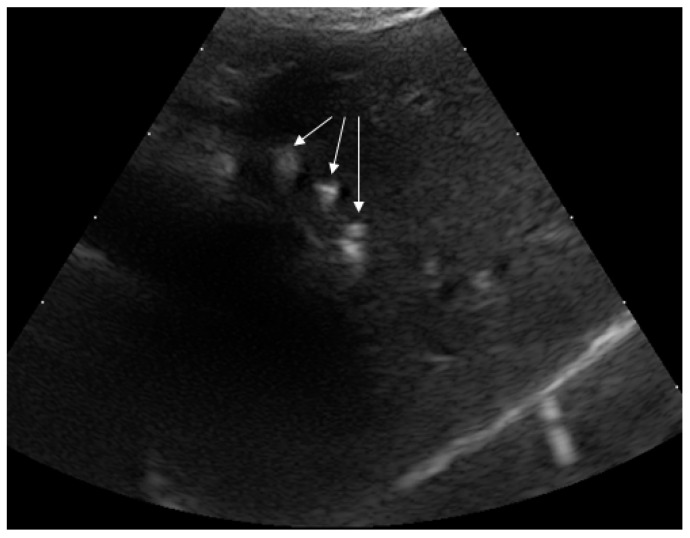
The US examination taken as a guide for the liver biopsy showed mineralizations within the liver parenchyma, most likely quadrate lobe (white arrows).

After the CT study an US-guided biopsy of the liver was carried out (MyLab Eight, Esaote SpA, Genoa, Italy) with no complications. The US examination, despite not complete, showed mineralization (Figure 2) of the liver lobes, as well as other findings seen in CT.

#### 2.4.3. Endoscopy

Gastroduodenoscopy showed gastric mucosal hyperemia and irregular mucosa of the duodenum, with squat and fused *villi* alternating with normal areas. Biopsies from the stomach and duodenum were taken for histopathological examination.

#### 2.4.4. Histopathology

**Figure 3:**
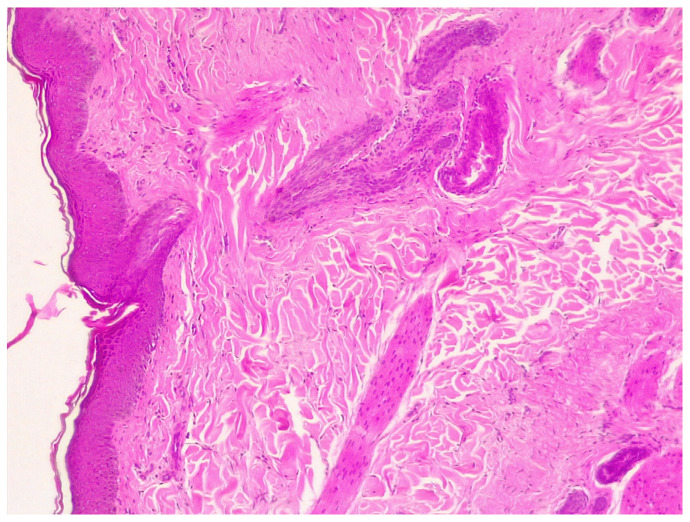
Skin histopathology: the epidermis showed moderate acanthosis and small numbers of inflammatory cells in the superficial dermis, composed by neutrophils, lymphocytes, and few plasma cells.

Liver histopathology revealed hypoplasia and hyperplasia of hepatic veins and arterioles, respectively. Gastric biopsy showed a mild lympho-plasmacellular gastritis while in the duodenum biopsy a mild lympho-plasmacellular duodenitis with fibrosis was observed. Skin biopsy revealed hyperplastic dermatitis. The epidermis had moderate acanthosis and small numbers of inflammatory cells in the superficial dermis, composed of neutrophils, lymphocytes, and few plasma cells (Figure 3). In one biopsy a fragmented intracorneal pustule was observed, with large numbers of degenerate neutrophils surrounded by lamellar keratin. The process was interpreted as superficial pyoderma.

#### 2.4.5. Molecular Analysis

Skin biopsy and blood samples were subjected to DNA extraction using commercial kits, i.e., Exgene Blood extraction kit and Exgene Tissue extraction kit (GeneAll Biotech, Seoul, South Korea), respectively, following the manufacturer’s instructions. A fragment of ~373 bp of the18S rRNA gene of *Hepatozoon* spp. was amplified using specific primers [19]. PCR reactions were carried out carried out in a 25 μL reaction mixture containing 2 μL of genomic DNA, 12.5 μL of Ready Mix REDTaq (Sigma, St. Louis, MO, USA), and 0.25 μL of each corresponding primer (50 μM). PCRs were performed in a thermal cycler (2700; Applied Biosystems, Foster City, CA, USA,) as previously described [21].

PCR Amplicons were sequenced for a species diagnosis. A convenient dataset (i.e., around 50% of the amplicons, with high quality and quantity of the PCR products) was purified using a QIAquick^®^ Gel Extraction Kit (Qiagen, GmbH, Hilden, Germany) and sequenced by a commercial laboratory (BMR-Genomics, Padova, Italy). Sequences were determined in both strands, aligned, and compared with those available in GenBank using the Basic Local Alignment Search Tool (BLAST: https://blast.ncbi.nlm.nih.gov/Blast, accessed on 5 April 2021), confirming the identity of *H. canis* both in the skin and blood samples.

## 3. Discussion

The present case report suggests a potential role of *H. canis* in a systemic disease in a puppy, adding possible new clinical knowledge on canine hepatozoonosis. To date, mostly subclinical *H. canis* infections have been reported in dogs and the severity of the disease has been correlated with the degree of the parasitaemia [22,23,24]. Most clinical signs herein detected fit with those previously reported, i.e., lethargy, fever, anorexia, weight loss, and gastrointestinal signs [1,12]. Though lameness is usually associated with *H. americanum* infection, this study and other findings suggest that a skeletal involvement could be more frequent than thought in dogs infected with *H. canis* [1,13].

Skin alterations are an uncommon finding in canine hepatozoonosis due to *H. canis*, as they have been reported only in few case reports [15,16,25]. In the one reported in New Jersey, the dog showed a raised, pruritic, alopecic, subcutaneous fluctuant swelling (2 cm in diameter) containing *H. canis* gamonts as the only alteration [15]. In this report, the skin lesions of the dog cannot be ultimately correlated to *H. canis*, as intralesional gamonts were not microscopically observed. Nevertheless, the exclusion of other causes (e.g., ectoparasites infestation, food/environment allergies, hepatocutaneous syndrome) and the detection of *H. canis* DNA in situ suggest the involvement of this protozoan in the pathogenesis of the skin lesions.

The laboratory findings were similar to those reported in previous *H. canis* infections. Mild normochromic anaemia is the most common laboratory finding [1,11,16] while higher leukocyte counts usually correspond to a higher parasitemia [1]. However, the lack of specificity of these alterations does not allow to attribute them unequivocally to *H. canis.*

The increased pre- and post-prandial bile acids level were consistent with portal systemic shunts, highlighted via the CT exam. Liver abnormalities were attributed to a pre-existing hepatic disease, as no parasitic stages were detected at the liver histopathological examination. Portal hypertension associated with *H. canis* has never been reported, though the merogony of *H. canis* occurs in the liver, and chronic hepatitis related to the presence of schizonts has been reported [18,26]. Thus, the role of *H. canis* in contributing to a pre-existing cholangiohepatitis, the portal hypertension, and/or its role in the worsening of a non-related hepatic disease cannot be definitively excluded.

With all likelihood, the 2-month-old dog of the present case acquired the infection via transplacental transmission, given the early onset of clinical signs. To date, no data is available on the possible effects of *H. canis* on the fetal development and in the pathogenesis of possible acquired hepatic disorders; thus, knowledge on vertically transmitted infections should be implemented.

Relapsing of clinical signs and/or failure of the therapeutic treatment have been frequently described in dogs with hepatozoonosis [27]. The negativization of the blood smear may be an inefficient criterion for the evaluation of treatment response and the follow up should be performed using molecular techniques [27,28]. The therapeutic management of canine hepatozoonisis is challenging as no drug is labelled for the treatment of canine hepatozoonosis in Europe and a lack of negativization has been observed also after prolonged off-label treatments [18]. New studies aiming at setting up adequate therapeutic guidelines are here advocated.

The relocation of dogs is one main driver fostering the emergence of vector-borne diseases, including hepatozoonosis, in previously free areas [17,29,30]. Accordingly, the dog of the present report was adopted from southern Italy, i.e., an endemic area for *H. canis* [5,28,31], and moved to an area of Northern Italy where this protozoan had never been described thus far. The uncontrolled movement of potentially infected dogs can be dangerous as *R. sanguineus*, the main vector of *H. canis*, is widespread in Northern Italy and in general in Europe, and present all year around [32,33]. Thus, improving the awareness on VBDs, including hepatozoonosis and the establishment of appropriate control programs (e.g., for animals moving from or to endemic areas) are pivotal to appropriately manage and control the spreading of the disease [34]. Regular and adequate prophylactic treatments are, in fact, often overlooked by owners. Thus, veterinarians should educate them on the correct management of dogs in terms of adequate timing, schedule and *spectrum* of action of products available on the market [34].

## 4. Conclusions

The role of *H. canis* in the systemic disease herein described is plausible as (i) skin lesions have been previously described in canine hepatozoonosis, (ii) pre-natal hepatic alterations due to a vertical transmission of *H. canis* can be considered and call for purposed investigations, and (iii) *H. canis* might have contributed to the worsening of a pre-existing hepatic disease not related to the infection. The pathogenic mechanisms elicited by *H. canis* should be further studied, with a particular focus on the vertical transmission route and its potential implications on the health of young animals, in which the infection is often more severe than in adult dogs.

## Figures and Tables

**Table 1:** Altered values of the haemato-biochemical, urine and coagulation analyses performed on Visit 1 (2 months old), Visit 2 (5 months old) and Visit 3 (10 months old).

Analyte	Value	Normal Range	Units
Visit 1			
HCT	34	37–55	%
WBC	17.75	6–17	10^9^/L
Neutrophils	14.60	3–12	10^9^/L
Basal cortisol	9.76	1–5	mcg/dL
Visit 2			
HCT	36.9	37–55	%
WBC	14.68	6–17	10^9^/L
Neutrophils	12.61	3–12	10^9^/L
Total protein	5.3	5.5–7.6	g/dL
Visit 3			
HCT	41.2	37–55	%
Platelets	489 × 10^9^	103–395	mcL
Albumin	2.01	2.4–3.8	g/dL
Total protein	5.3	5.5–7.6	g/dL
Cobalamin	<150	251–908	ng/L
Folates	6.60	7.7–24	mcg/L
ALP	875	20–120	IU/L
ALT	475	15–64	IU/L
AST	166	12–54	IU/L
TLI	>50	5.2–35	mcg/L
Total iron	69	76–173	mcg/dL
Pre-prandial bile acids	53.55	0–22	μmol/L
Post-prandial bile acids	97.47	0–30	μmol/L
Bilirubinuria	1	<1	mg/dL
Urobilinogen	4	<1	mg/dL
aPTT	108	75–105	sec

## Data Availability

All the data generated are described in the paper.
